# Assessment of myocardial function in Retrievers with dilated cardiomyopathy using 2D speckle tracking echocardiography: a pilot study

**DOI:** 10.3389/fvets.2024.1462437

**Published:** 2024-12-05

**Authors:** Lina Hamabe, Kazumi Shimada, Miki Hirose, Mizuki Hasegawa, Aki Takeuchi, Tomohiko Yoshida, Daigo Azakami, Ahmed S. Mandour, Ryou Tanaka

**Affiliations:** ^1^Department of Veterinary Medicine, Faculty of Agriculture, Tokyo University of Agriculture and Technology, Tokyo, Japan; ^2^Department of Clinical Veterinary Medicine, Obihiro University of Agriculture and Veterinary Medicine, Obihiro, Hokkaido, Japan; ^3^Department of Animal Medicine (Internal Medicine), Faculty of Veterinary Medicine, Suez Canal University, Ismailia, Egypt

**Keywords:** 2D-STE, canine dilated cardiomyopathy, dog, echocardiography, Retrievers, strain analysis

## Abstract

Early diagnosis of canine dilated cardiomyopathy (DCM) is complicated by the presence of a prolonged asymptomatic phase, for which a comprehensive evaluation of myocardial function is essential. This pilot study was conducted to evaluate the myocardial function in dogs with DCM using two-dimensional speckle tracking echocardiography (2D-STE). Nine client-owned Retrievers with DCM and twelve client-owned clinically normal Retrievers were comparatively evaluated using standard echocardiography and 2D-STE. Dogs with DCM were characterized by significant dilation of the left ventricle (LV), thinning of the LV wall, and myocardial hypokinesis when compared to clinically normal dogs. The global strain analysis showed a significant reduction of strain in both radial and circumferential directions, and the regional strain analysis revealed a greater degree of myocardial dysfunction at the LV free wall in the circumferential direction in dogs with DCM. The regional strain analysis also demonstrated a difference in the pattern of contraction between dogs with DCM and clinically normal dogs. The results of this study illustrate the ability of 2D-STE to evaluate both global and regional myocardial function in dogs with DCM and show differences between dogs with DCM and clinically normal dogs.

## 1 Introduction

Canine dilated cardiomyopathy (DCM) is the most common form of cardiomyopathy in dogs (1, 2). It is characterized by progressive ventricular dilation resulting from the loss of myocardial contractility in the absence of any other cardiac, pulmonary, or systemic disease that may lead to similar characteristics (1–3). While there are different phenotypes of DCM in dogs, idiopathic DCM is often considered breed-specific and is most often seen in large and giant breed dogs (1–3). There is a prolonged duration of the disease’s asymptomatic, pre-clinical phase without any associated clinical signs, which is known to last for months to years (1, 2). Gradual progression eventually leads to the symptomatic clinical phase, which is associated with the presence of congestive heart failure (CHF) or sudden death (1, 4). While the importance of early disease detection is well acknowledged, identifying this pre-clinical phase has proven difficult (1, 4).

Standard echocardiography is a widely accepted method for evaluating myocardial function. It has commonly been used to assess myocardial changes observed with DCM, and the diagnosis is made based on echocardiographic evidence of left ventricular (LV) dilation, myocardial hypokinesis, and increased sphericity (1, 2, 5, 6). LV fractional shortening (FS) is the most commonly used echocardiographic measurement of systolic function; however, it does not reflect the true systolic function because it only assesses myocardial movements in locally restricted segments (6, 7). Additionally, FS is affected by the loading conditions and the presence of mitral insufficiency, which further limits its usefulness (6, 8). Simpson’s method of discs is another method that can assess myocardial changes, which uses two-dimensional measurements to estimate volume measurements (9). While it may provide more accurate volume measurements, it does not allow regional assessment. Similarly, E-point to septal separation, which measures the distance between the maximal early diastolic motion (E-point) of the septal mitral valve leaflet to the interventricular septum (IVS), is another commonly used parameter of systolic function. However, its measurement is restricted to a local segment and is influenced by the presence of mitral stenosis and aortic regurgitation (4, 8). Since quantitative assessment of regional myocardial function cannot be adequately made, standard echocardiography may not be particularly sensitive in detecting subtle myocardial changes observed in the early asymptomatic phase of DCM nor in monitoring mild disease progression (5).

Two-dimensional speckle tracking echocardiography (2D-STE) is an advanced echocardiographic technique that assesses myocardial function by quantifying myocardial deformation (10). Strain, the parameter of 2D-STE, quantifies the myocardial deformation as the percentage change in the length of the myocardium relative to its original length during systole and diastole, and it allows quantitative evaluation of both global and regional myocardial function (10, 11). LV myocardium is divided into segments, where regional strain is the average value within each segment, and global strain is the average value of all segments, representing the overall myocardial function (10, 12). In addition, segmental analysis allows the quantification of LV synchronicity by identifying variability between the segments (10, 13). In humans, reduced strain values are seen in patients with DCM, and 2D-STE has been shown to be useful in predicting the outcome of heart transplantation and cardiac resynchronization therapy in patients with DCM (14–17).

This pilot study aimed to illustrate the ability of 2D-STE to comprehensively evaluate the myocardial function in dogs with DCM by quantifying both global and regional myocardial function and determining whether it allows distinction from clinically normal dogs.

## 2 Materials and methods

### 2.1 Study population

Client-owned Retrievers diagnosed with DCM (DCM group) and clinically normal Retrievers (control group) were recruited for enrollment in this study. DCM group consisted of nine Retrievers presented at the Tokyo University of Agriculture and Technology Animal Medical Centre for the diagnosis and treatment of DCM and was diagnosed as clinical DCM with or without the presence of symptoms of CHF. Diagnosis was made based on echocardiographic evidence of LV dilation (LVIDdN >1.7), increased sphericity, and depressed systolic function (FS < 25%), and with the presence of at least one of the following conditions: (1) radiographic evidence of left-sided or biventricular cardiac enlargement, (2) radiographic evidence of pulmonary oedema or pleural effusion, and (3) electrocardiographic (ECG) evidence of arrhythmia including atrial fibrillation, ventricular premature complexes and ventricular tachycardia (1, 5, 18). Comprehensive cardiovascular examinations including echocardiography, radiography, ECG, and oscillometric blood pressure measurements were performed to exclude any congenital or acquired cardiac disease and systemic hypertension in addition to DCM. Blood tests consisting of a complete blood count, serum biochemistry, and thyroid panel, including serum thyroxine, serum free thyroxine, and endogenous canine thyrotropin concentrations, were performed to exclude any concurrent systemic disease that may affect cardiac function, unless the referring veterinarians had done them within two-weeks of referral. Owners were questioned regarding dietary history to exclude the possibility of grain-free diet, metabolic deficiencies and history of use of drugs known to affect cardiac function. Dogs with echocardiographic data from the previous examinations that allowed a definitive diagnosis of DCM were allowed to enroll during the medical treatment.

Clinically normal Retrievers included 12 dogs with an unremarkable history and normal physical examination, with no evidence of congenital or acquired cardiac disease seen with standard echocardiographic examination. Dogs in the control group were selected based on breed, sex, age, and bodyweight to match the DCM group.

### 2.2 Study protocol

The study was carried out in compliance with the guidelines established by the Tokyo University of Agriculture and Technology Animal Medical Center, with informed consent obtained from all owners. Echocardiographic evaluations, including standard echocardiography and 2D-STE, were performed using either of the ultrasonography units: ALOKA prosound *α* 10 equipped with a 3–8 MHz phased array transducer probe (UST-52108) (Hitachi Aloka Medical, Ltd., Japan) and LISSENDO 880LE equipped with a 9–12 MHz phase array transducer probe (S31) (Fujifilm Ltd., Japan). A mean of at least three measurements was obtained from consecutive cardiac cycles in sinus rhythm for each parameter. The investigators were aware of the clinical status of the dogs as evaluations took place in a clinical setting. None of the dogs were sedated during the echocardiography.

### 2.3 Standard echocardiography

The examination was performed in accordance with the published methodology in veterinary literature (6). The dimensions of the LV chamber were measured using M-mode from the right parasternal short-axis view. The measurements included LV internal dimension at end-diastole (LVIDd) and at end-systole (LVIDs), interventricular septal (IVS) thickness at end-diastole (IVSd) and at end-systole (IVSs) and LV free-wall (LVFW) thickness at end-diastole (LVFWd) and at end-systole (LVFWs), measured at the level of the papillary muscle. Diastolic and systolic LVID normalized to body weight (LVIDdN and LVIDsN, respectively) were calculated as reported by Cornell et al. (18). LV systolic function was evaluated using FS and systolic time intervals. FS was calculated using the following equation: FS (%) = LVIDd-LVIDs/LVIDd x 100. Peak pulmonary blood flow velocity (PA Vmax) and peak aortic blood flow velocity (Ao Vmax) were obtained using the spectral Doppler from the right parasternal short-axis view at the level of the pulmonary artery and left parasternal apical five-chamber view, respectively. Systolic time intervals, which are the ratio of pre-ejection period and ejection time (PEP:ET) and stroke volume (SV), were measured from spectral Doppler aortic velocity. LV diastolic function was assessed by trans-mitral rapid ventricular filling (E) and atrial contraction (A), and E to A ratio (E/A) obtained from the trans-mitral flow profile at the left parasternal apical four-chamber view. The left parasternal apical four-chamber view was used for the pulse-wave tissue Doppler imaging (TDI) assessment to measure the mitral annular tissue velocities. Systolic (S′) and early (E′) diastolic myocardial velocities were obtained at IVS and LVFW, and the corresponding ratio between E and E′ (E/E′) at IVS and LVFW was calculated.

### 2.4 Two-dimensional speckle tracking echocardiography

Right parasternal short axis view at the level of the papillary muscle with the frame rate of 70–110 frames/s was acquired, which was then analyzed offline (DAS-RS1 software 6.0v, Hitachi Aloka Medical, Ltd., Japan). Firstly, the endocardial and epicardial borders of the LV were manually traced at end-systole by placing several regions of interest (ROIs), which were then automatically tracked on a frame-by-frame basis by the software. Strain measurements were taken in the radial and circumferential directions. Six-segment model was used for the regional analysis, where the LV was divided into six segments (anterior (AN), lateral (LT), posterior (PS), inferior (IN), septal (SP), and anterior septal (AS)). LV synchrony was assessed by calculating the synchrony time index (STI), which is the difference in timing of peak strains from the earliest to the latest segments. Strain parameters were obtained, including global peak strain in the radial and circumferential directions (GRS and GCS), regional strains at the six segments in both directions, and STI in the radial direction.

### 2.5 Statistical analysis

Statistical analysis was performed using statistical software (Prism 8.0v, GraphPad Software Inc., USA). Normal distribution was graphically inspected and tested using the Shapiro–Wilk test. Significant differences between the DCM and control groups were evaluated using unpaired Student’s t-test for normally distributed parameters and Mann–Whitney test for parameters not normally distributed. A significant difference was defined as *p* < 0.05.

## 3 Results

### 3.1 Study population

Dogs in the DCM group (*n* = 9) consisted of three Golden and six Labrador Retrievers, of which three were male and six were female. The average age and bodyweight for the DCM group were 11.33 ± 2.35 years old (range 6–14 years old) and 25.88 ± 4.88 kg (range 16.00–32.15 kg). The control group (*n* = 12) consisted of six Golden and six Labrador Retrievers, four of which were male and eight of which were female. The average age and bodyweight for the control group were 9.50 ± 1.83 years old (range 6–12 years old) and 26.90 ± 5.53 kg (range 18.30–38.60 kg). There was no statistical difference with age (*p* = 0.06) and bodyweight (*p* = 0.66) between the groups.

While four dogs were asymptomatic, the remaining five dogs from the DCM group were presented with one or more of the following clinical signs of CHF: cough (*n* = 2), exercise intolerance (*n* = 4), and ascites (*n* = 2). ECG abnormality of isolated ventricular premature beats was observed in two dogs. Among five dogs presented with clinical signs, four dogs were already being treated with one or a combination of the following agents: angiotensin converting enzyme inhibitor (*n* = 3), furosemide (*n* = 3), pimobendan (*n* = 3), and taurine (*n* = 1).

### 3.2 Standard echocardiography

Table 1 shows the results of standard echocardiography of Retrievers with DCM and clinically normal Retrievers. Standard echocardiographic evaluation of the control group revealed all parameters to be within the reference range (6, 19–22). On the other hand, in the DCM group, LVIDd was above, and FS was below the reference range (6, 19–22). Compared to the control group, the DCM group showed statistically significant dilation of LV (LVIDs *p* = 0.0009) and thinning of LV walls (IVSs *p* = 0.0114, LVFWd *p* = 0.0173, LVFWs *p* = 0.0088), except for IVSd and LVIDd. LVIDdN and LVIDsN also revealed significant differences between the groups (*p* = 0.0269 and *p* = 0.0024, respectively). Additionally, significant reductions in FS (*p* < 0.0001) and S′ IVS (*p* = 0.0002), and an increase in Ao PEP/ET (0.0210) were observed in the study group compared to the control group, suggestive of impaired systolic function in the DCM group. A significant reduction in Ao Vmax (*p* = 0.0210) and SV (*p* = 0.0441) in the DCM group was also observed. Moreover, mitral valve regurgitation secondary to mitral annulus dilation was observed in three dogs in the DCM group that also showed signs of CHF. The average heart rate during the echocardiographic examination was 104.40 ± 19.80 beats per minute (bpm) (range 80.00–134.00 bpm) for the DCM group and 108.90 ± 14.05 bpm (range 84.00–130.00 bpm) control group, showing no significant differences between the groups (*p* = 0.57).

**Table 1:** Standard echocardiographic parameters of Retrievers with DCM and clinically normal Retrievers.

	DCM (*n* = 9)	Control (*n* = 12)	*p*-Value
IVSd (mm)	8.67 ± 2.01	9.74 ± 1.39	0.165
IVSs (mm)	11.05 ± 2.74	14.03 ± 2.15	0.0114
LVIDd (mm)	49.21 ± 12.92	40.69 ± 3.77	0.0722
LVIDs (mm)	37.00 (28.50–70.06)	27.18 (21.33–31.56)	0.0009
LVFWd (mm)	8.07 ± 1.21	9.96 ± 1.91	0.0173
LVFWs (mm)	11.10 ± 1.44	13.61 ± 2.24	0.0088
LVIDdN	1.90 ± 0.49	1.55 ± 0.12	0.0269
LVIDsN	1.44 ± 0.46	0.98 ± 0.08	0.0024
FS (%)	19.35 ± 6.05	32.47 ± 4.54	<0.0001
PA Vmax (cm/s)	56.75 ± 18.95	69.50 ± 16.92	0.1325
Ao Vmax (cm/s)	64.67 ± 21.34	99.25 ± 31.86	0.0210
Ao PEP:ET	0.43 ± 0.09	0.29 ± 0.07	0.0025
SV (ml)	8.10 (5.80–17.00)	11.52 (8.30–24.00)	0.0441
E (cm/s)	53.55 ± 25.78	56.36 ± 8.79	0.7308
A (cm/s)	40.52 ± 7.84	48.32 ± 9.58	0.0862
E/A	1.35 ± 0.62	1.22 ± 0.31	0.5602
S′ IVS (cm/s)	5.31 ± 1.71	10.51 ± 2.55	0.0002
E/E′ IVS	10.45 (6.06–29.10)	7.06 (5.13–11.55)	0.0831
S′ LVFW (cm/s)	8.66 ± 3.27	10.43 ± 1.51	0.1577
E/E′ LVFW	8.93 ± 4.42	5.43 ± 1.35	0.0245

DCM, dilated cardiomyopathy; SD, standard deviation; IVSd, interventricular septal thickness at end-diastole; IVSs, interventricular septal thickness at end-systole; LVIDd, left ventricular internal diameter at end-diastole; LVIDs, left ventricular internal diameter at end-systole; LVFWd, left ventricular free wall thickness at end-diastole; LVFWs, left ventricular free wall thickness at end-systole; LVIDdN, diastolic left ventricular internal diameter normalized to body weight; LVIDsN, systolic left ventricular internal diameter normalized to body weight; FS, fractional shortening; PA Vmax, peak pulmonary blood flow velocity; Ao Vmax, peak aortic blood flow velocity; Ao PEP/ET, aortic pre-ejection period to left ventricular ejection time ratio; SV, stroke volume; E, rapid ventricular filling; A, atrial contraction; E/A, ratio of rapid ventricular filling to atrial contraction; S′ IVS, systolic myocardial velocity at interventricular septum; E/E′ IVS, ratio of rapid ventricular filling to rapid diastolic motion at interventricular septum; S′ LVFW, systolic myocardial velocity at left ventricular posterior wall; E/E′ LVPW, ratio of rapid ventricular filling to rapid diastolic motion at left ventricular free wall.

### 3.3 Two-dimensional speckle tracking echocardiography

**Figure 1:**
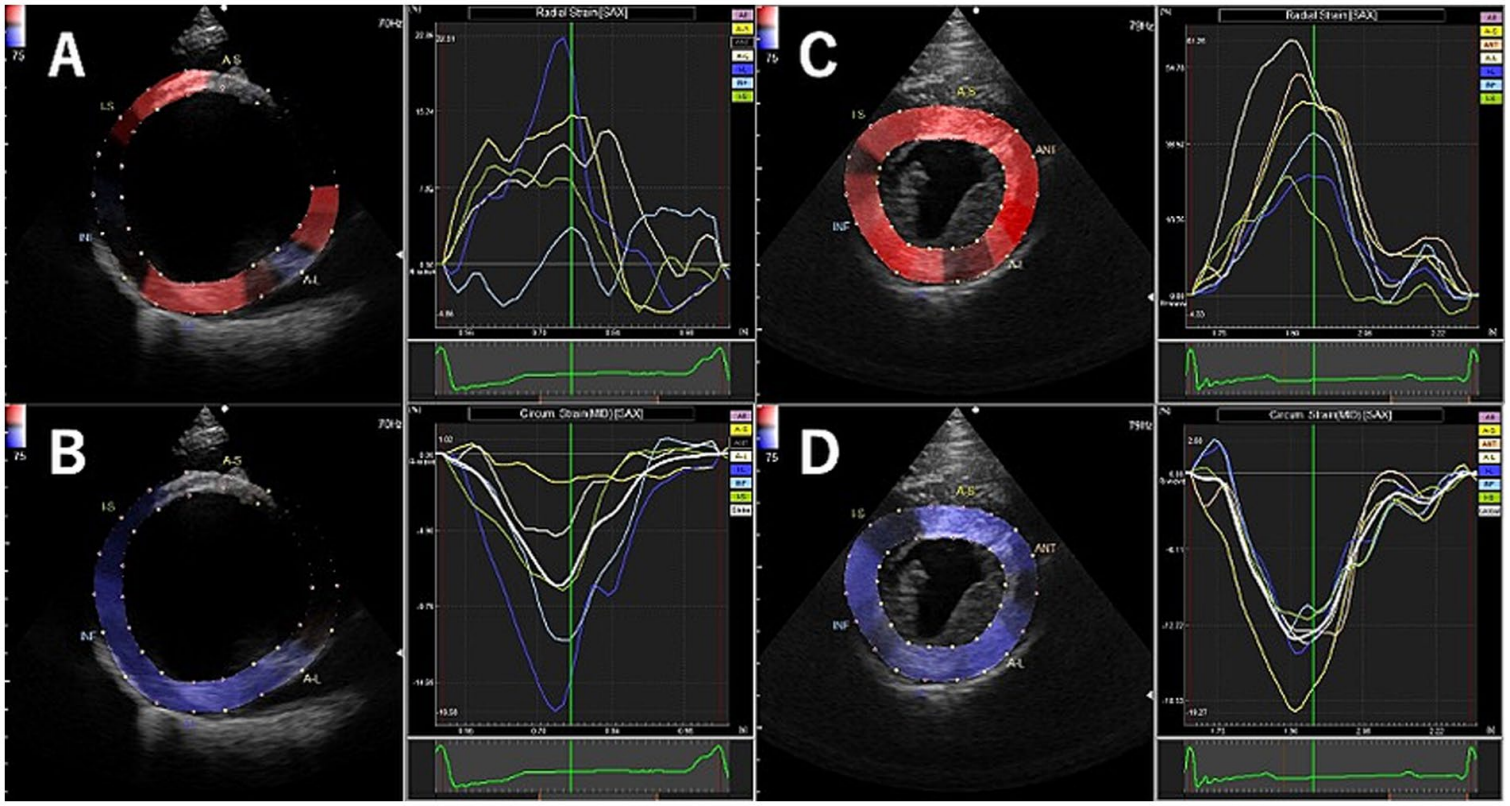
Examples of radial and circumferential strain profiles obtained from right parasternal short axis view at the level of the papillary muscle using two-dimensional tissue tracking in Retrievers with DCM (A: radial, B: circumferential) and clinically normal Retrievers (C: radial, D: circumferential).

**Figure 2:**
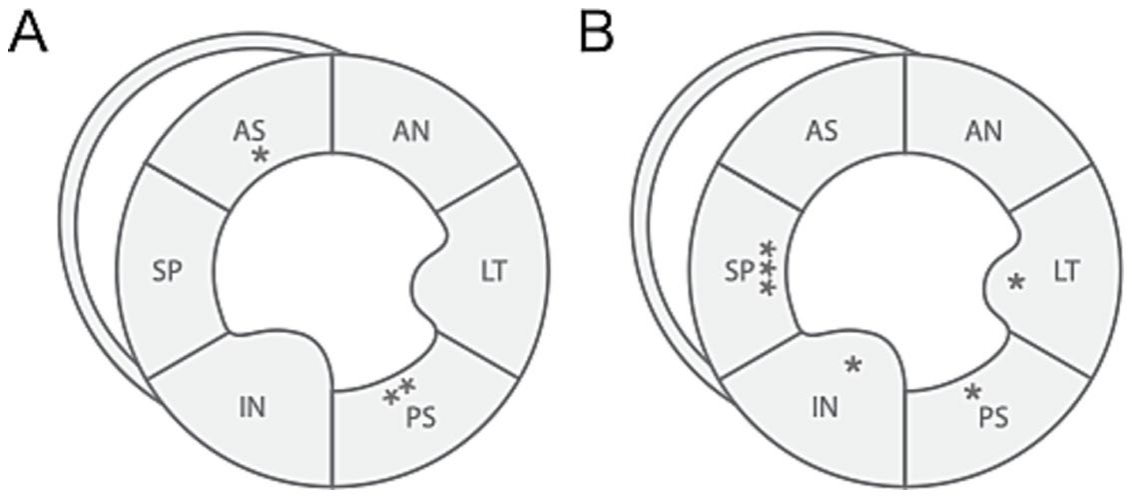
Illustration of differences in regional strain values between Retrievers with DCM and clinically normal Retrievers observed in (A) radial and (B) circumferential directions. *p < 0.05, **p < 0.005, ***p < 0.001. AN, anterior; LT, lateral; PS, posterior; IN, inferior; SP, septal; AS, anterior septal.

**Figure 3:**
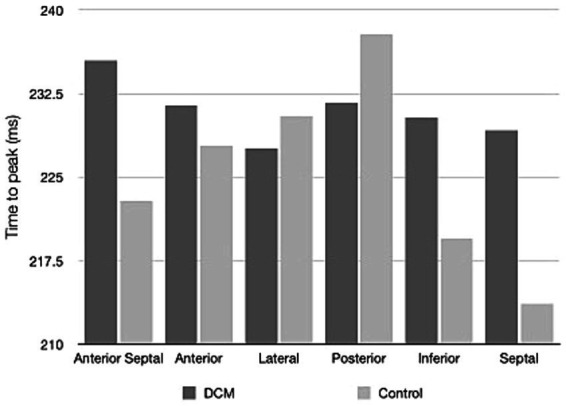
Time to peak values for the regional myocardial segments in radial direction illustrating the pattern of contraction of Retrievers with DCM and clinically normal Retrievers.

Table 2 shows the results of 2D-STE of Retrievers with DCM and clinically normal Retrievers, and Figure 1 shows examples of radial and circumferential strains for Retrievers with DCM and clinically normal Retrievers. Comparison of 2D-STE values between groups revealed global strain values to be significantly lower in the DCM group in both radial and circumferential directions (GRS *p* = 0.0401, GCS *p* = 0.0026). Regionally, significantly lower strain values were observed with the DCM group at the anterior septal and posterior segments in radial direction (AS *p* = 0.0236, PS = 0.0054), and at lateral, posterior, inferior and septal segments in circumferential directions (LT *p* = 0.0252, PS *p* = 0.0157, IN *p* = 0.0118, SP *p* = 0.0004) (Figure 2). Evaluation of the time to peak for each myocardial segment at the radial direction showed a homogenous pattern of contraction with the earliest time to peak at the septal segment in the control group. In contrast, the DCM group showed heterogenous pattern of contraction with the earliest time to peak at the lateral segment (Figure 3). Lastly, there was no significant difference in STI between groups.

**Table 2:** Two-dimensional speckle-tracking echocardiographic parameters of Retrievers with DCM and clinically normal Retrievers expressed as mean ± SD.

	DCM (*n* = 9)	Control (*n* = 12)	*p*-Value
Global radial strain (%)	21.62 ± 9.46	32.24 ± 11.88	0.0401
Regional radial anterior septal strain (%)	19.55 ± 13.14	31.64 ± 9.43	0.0236
Regional radial anterior strain (%)	22.90 ± 12.76	27.60 ± 14.46	0.4479
Regional radial lateral strain (%)	22.50 ± 12.56	33.33 ± 16.56	0.1181
Regional radial posterior strain (%)	20.01 ± 10.57	36.33 ± 12.62	0.005
Regional radial inferior strain (%)	21.52 ± 10.52	32.48 ± 15.93	0.09
Regional radial septal strain (%)	23.24 ± 7.45	32.04 ± 14.37	0.1114
Global circumferential strain (%)	−8.60 ± 2.92	−12.34 ± 2.04	0.0026
Regional circ. anterior septal strain (%)	−10.35 ± 5.18	−13.52 ± 4.13	0.1351
Regional circ. anterior strain (%)	−8.54 ± 4.55	−10.62 ± 3.02	0.2228
Regional circ. lateral strain (%)	−8.20 ± 3.93	−12.15 ± 3.50	0.0252
Regional circ. posterior strain (%)	−9.34 ± 4.86	−13.90 ± 3.02	0.0157
Regional circ. inferior strain (%)	−7.17 ± 2.79	−10.55 ± 3.57	0.0118
Regional circ. septal strain (%)	−8.11 ± 2.17	−13.44 ± 3.20	0.0004
STI (ms)	45.37 ± 18.80	47.52 ± 19.50	0.8021

DCM, dilated cardiomyopathy; SD, standard deviation; STI, synchrony time index.

## 4 Discussion

This pilot study illustrated the difference in myocardial function between nine Retrievers with DCM and 12 clinically normal Retrievers with comparable sex, age, and body weight. In this study, Golden and Labrador Retrievers were collectively grouped as Retrievers as they are known to be closely related based on genetic and phenotypic breed grouping (23).

Both standard echocardiography and 2D-STE analysis revealed significant differences between the DCM and the control groups, even though the DCM group included asymptomatic dogs and dogs already receiving medical treatment. In standard echocardiography, dogs with DCM showed significant dilation and thinning of the LV and myocardial hypokinesis, illustrated by the significant increase in LVIDs, LVIDdN and LVIDsN, and significant reduction in LV wall thickness and parameters of systolic function in comparison to the clinically normal dogs. The lack of significant difference in LVIDd between the groups is thought to be due to the small sample size and the greater variability among the DCM group. Three dogs in the DCM group had mild to moderate mitral valve regurgitation secondary to the mitral annulus dilation in association with the dilation of LV. Additionally, dogs with DCM showed a significantly higher value of E/E’ at the LVFW, which was suggestive of volume overload, as E/E’ is known to be a good indicator of increased LA pressure (24).

Strain analysis is based on deformation imaging where strain parameters are calculated from 2D displacement of the myocardium (10, 11). Therefore, unlike the tissue-Doppler analysis, 2D-STE is considered angle-independent, allowing the myocardial function to be evaluated from various spatial orientations (10, 11). A comparison of global analysis between the groups showed significantly lower strain values in dogs with DCM in both radial and circumferential directions, indicating significant myocardial dysfunction. Myocardial contractility occurs in both radial and circumferential directions, and radial and circumferential strains are sensitive indicators of myocardial contractility in dogs (10, 25). It is known that radial deformation indicates transmural dysfunction, whereas circumferential deformation indicates subendocardial and subepicardial dysfunction (26). Therefore, the results of this study suggest that the myocardial dysfunction of DCM involves the entire myocardium. Previous studies in humans and dogs have reported that myocardial contractility primarily occurs in the radial and circumferential directions, and that deformation in these directions may serve as a sensitive indicator of myocardial contractility (25, 27, 28).

One of the major advantages of 2D-STE includes the ability to allow simultaneous evaluation of the global and regional myocardial function (10, 11). Regional analysis of 2D-STE revealed that in the circumferential directions, significantly lower strain values were focused around the LVFW (including the lateral, posterior, inferior and septal segments). Such result suggests that at least in the endocardium or the epicardium of the LV, LVFW is more susceptible to myocardial dysfunction associated with DCM. In normal LV, contraction occurs in homogenous pattern with the septal segment contracting slightly earlier than the lateral and posterior segments (14). A similar result was observed in the clinically normal dogs, where on average the septal segment was the first and lateral and posterior segments were the last to reach peak strain. In contrast, on average, in dogs with DCM, the lateral segment was the first and the anterior septal segment was the last to reach peak strain, showing heterogeneous pattern of contraction. It is known that a heterogeneous pattern of contraction ultimately results in reduced SV (14). In this study, a significant reduction of SV was observed in the dogs with DCM, which suggests that the heterogeneous pattern of contraction may indicate myocardial dysfunction, resulting in reduced SV. This point should further be studied under a condition of increased myocardial demands, for example using an exercise intolerance test. DCM has a prolonged period of asymptomatic phase during which myocardial hypokinesis may be the only detectable evidence (1, 2, 5). Therefore, the evaluation of the pattern of contraction may be used as additional criteria for the evaluation of DCM.

In human medicine, LV mechanical dyssynchrony is an important prognostic factor in patients with DCM (14). The orientation of the cardiac motion is largely radial and circumferential, and a reduction in systolic function is associated with increased radial mechanical dyssynchrony in patients with DCM (14, 17). However, this study did not show any significant difference in STI between groups, regardless of the demonstration of heterogeneous pattern of contraction in dogs with DCM. The proposed normal range for the STI in dogs is 0–45 ms (29). Interestingly, in this study, the STI value for the control group was just above the proposed normal range of STI, and for the DCM group, it was in the upper range. A similar study with clinically healthy Retrievers also showed a higher STI value in comparison to the proposed STI values (22). The higher STI values for the control and DCM groups in this study may be due to the sample population’s older age compared to the population of the proposed STI values, whose mean age was 3.1 years old. A study by Lopez-Alvarez has also evaluated the mechanical synchrony in Doberman Pinschers with DCM using TDI, which failed to show differences between the normal dogs and dogs with DCM, a result similar to this study (30). Mechanical dyssynchrony may be less evident in dogs with DCM.

The radial and circumferential strains can be evaluated in parasternal short-axis views at the basal, papillary muscles and apex level of the LV. In both humans and dogs, strain values appear greater at the apex in comparison to the base creating an apex to base gradient (31–33). In a case with dogs with pre-clinical DCM, a study by Predo et al. showed that, while overall decrease in radial and circumferential strains were observed at all three levels of the LV, the greatest difference was observed at level of the papillary muscles (30). Based on such finding, in this study, strain analysis was only performed at the level of the pupillary muscle, none the less, a further study including the analysis at all three level should be conducted.

There are several limitations to this study. As this study was a pilot study, the total number of dogs included in the study was small, and although the difference was statistically insignificant, the average age of DCM group was slightly older than the control group. Additionally, the DCM group included dogs with both asymptomatic and symptomatic DCM. In order to evaluate the true potential of 2D-STE to diagnose DCM at an early stage, ideally, the study should consist of three groups, including clinically normal dogs, dogs with asymptomatic DCM and symptomatic DCM. Further studies need to be conducted on a larger scale. Furthermore, some of the dogs that exhibited symptoms were already receiving medications including angiotensin converting enzyme inhibitor, furosemide and pimobendan. While these medications are essential for managing DCM, they may influence the myocardial function, particularly by improving systolic function and may later strain parameters. This presents a potential limitation of the study, as controlling for the effects of treatment on echocardiographic evaluation was not feasible. One of the advantages of 2D-STE is that it is angle-independent and can be analyzed in multiple spatial orientations. Since this study only included the analysis in radial and circumferential directions, longitudinal analysis should also be performed. Lastly, 2D-STE analysis has its own limitations, such as the additional cost of the software and the expertise and time required to perform the analysis after examinations.

This study evaluated the myocardial function using 2D-STE, and demonstrated that the strain parameters differed between dogs with DCM and clinically normal dogs. The results of global strain analysis indicate that dogs with DCM are associated with a significant reduction of strain values in both radial and circumferential directions. Regional strain analysis suggested the possibility of increased susceptibility of the LVFW to circumferential myocardial dysfunction. Moreover, dogs with DCM showed a heterogeneous pattern of contraction, which may be associated with the progression of myocardial dysfunction. Irrespective of the heterogeneous pattern of contraction in dogs with DCM, significant mechanical dyssynchrony was not observed.

2D-STE Evaluation of myocardial function using 2D-STE provides valuable information on both global and regional myocardial function and to quantify myocardial synchronicity, which is not readily possible with standard echocardiography. Additional information provided by the 2D-STE analysis allows for a comprehensive and accurate evaluation of myocardial function, which is essential for diagnosing myocardial dysfunction and monitoring treatment and may serve as a prognostic indicator.

## Data Availability

The original contributions presented in the study are included in the article/supplementary material, further inquiries can be directed to the corresponding author.
